# Intraoperative indocyanine green-visualization in a difficult to localize central cholangiocarcinoma – A case report

**DOI:** 10.1016/j.ijscr.2021.105973

**Published:** 2021-05-12

**Authors:** Till M. Hempfing, Daniela B. Husarik, Thomas Steffen

**Affiliations:** a Department of anaesthesiology and intensive care medicine, Hospital of the Canton of St. Gallen, CH-9007 St. Gallen, Switzerland; b Department of radiology and nuclear medicine, Hospital of the Canton of St. Gallen, CH-9007 St. Gallen, Switzerland; c Department of surgery, Hospital of the Canton of St. Gallen, CH-9007 St. Gallen, Switzerland

**Keywords:** CEUS, contrast enhanced ultrasonography, CT, computed tomography, ERCP, endoscopic retrograde cholangiopancreaticography, ICG, indocyanine green, MRI, magnetic resonance imaging, MRCP, magnetic resonance cholangiopancreaticography, Indocyanine green, Fluorescence, Liver tumour, Cholangiocarcinoma, Case report

## Abstract

**Introduction and importance:**

Near infrared fluorescence imaging with indocyanine green (ICG) can facilitate the intraoperative tumour localization and therefore a complete resection. Cholangiocarcinoma is an aggressive tumour and complete resection improves the outcome. Therefore, it is necessary to localize the tumour exactly but the translation of the preoperative imaging into the intraoperative setting can be difficult based only on sonography, computed tomography or magnetic resonance imaging.

**Case presentation/clinical findings and investigations/interventions and outcome:**

In this case a hepatic lesion suspicious for cholangiocarcinoma was discovered accidentally. Further diagnostics were unable to prove the diagnosis, therefore right hepatectomy was recommended and performed. Preoperatively ICG was administered and near infrared imaging was used intraoperatively clearly localizing the tumour, thus facilitating the resection. The intra- and postoperative course was uneventful.

**Relevance and impact:**

This case report supports the very promising intraoperative use of fluorescence imaging for the localization of superficial hepatic tumours. Timing and correct administration of ICG is important.

## Highlights

• Indocyanine green facilitates intraoperative tumour localization and improves outcome.
• Cholangiocarcinoma is an aggressive tumour with poor outcome.
• Surgery with a negative resection margin improves outcome.

## 1 Introduction

### 1.1 Background

Cholangiocarcinoma is an aggressive entity that accounts for about 20% of primary hepatic malignancies [1]. The incidence of intrahepatic biliary tumours is rising [2]. Due to the oligosymptomatic nature of biliary tumours only one third of the patients qualify for a curative treatment at the time of diagnosis, accounting for an overall 5-year survival rate of 10% [3] Surgical resection with negative resection margins is the only curative treatment and improves the 5-year survival rate up to 40% [4]. While tumours can usually be localized with preoperative imaging such as ultrasound, computed tomography scan (CT), or magnetic resonance imaging (MRI), exact localization of the tumours intraoperatively can be very challenging.

Since the 1960's indocyanine-green (ICG) has been established in assessing the liver function as it is exclusively metabolized in the hepatic parenchymal cells. ICG emits light at a wave length of around 835 nm when illuminated with near-infrared light and can therefore be used in fluorescence guided imaging with a tissue penetration up to 1 cm [5]. Combining these properties ICG has been established in hepatic surgery to help visualizing intrahepatic tumours and biliary structures intraoperatively [[6], [7], [8], [9]]. This technique has shown to improve the outcome after resection of both primary and metastatic liver tumours [10]. In hepatocellular carcinoma patterns of fluorescence have been associated with grade of differentiation: moderately or poorly differentiated tumours showed a lower liver-to-lesion contrast when stained with ICG [8,11].

### 1.2 Rationale

ICG staining in hepatic surgery is a trending research topic and guidelines on the use of intraoperative fluorescence are being established. There is an ongoing debate about the exact mode of application of ICG. To support current research we present a case in which intraoperative ICG was used to localize a central intrahepatic cholangiocarcinoma in accordance to the recommendation by Chen et al [7]

### 1.3 Guidelines and literature

Feasibility and effectiveness of intraoperative ICG staining in hepatic surgery has been evaluated since 2008 [6,8,12,13]. In 2020 a consensus guideline has been published on the use of ICG in hepatic surgery [5].

This work has been reported in line with the SCARE criteria [14].

## 2 Case report

### 2.1 Patient information

In a 62-year-old Caucasian male patient routine MRI follow-up was conducted yearly due to complex renal cysts. Beside the renal cysts the patient was treated for arterial hypertension with a combination of three antihypertensive drugs. His liver was steatotic, otherwise the patient was healthy.

### 2.2 Clinical findings/timeline/diagnostic assessment and interpretation

**Fig. 1:**
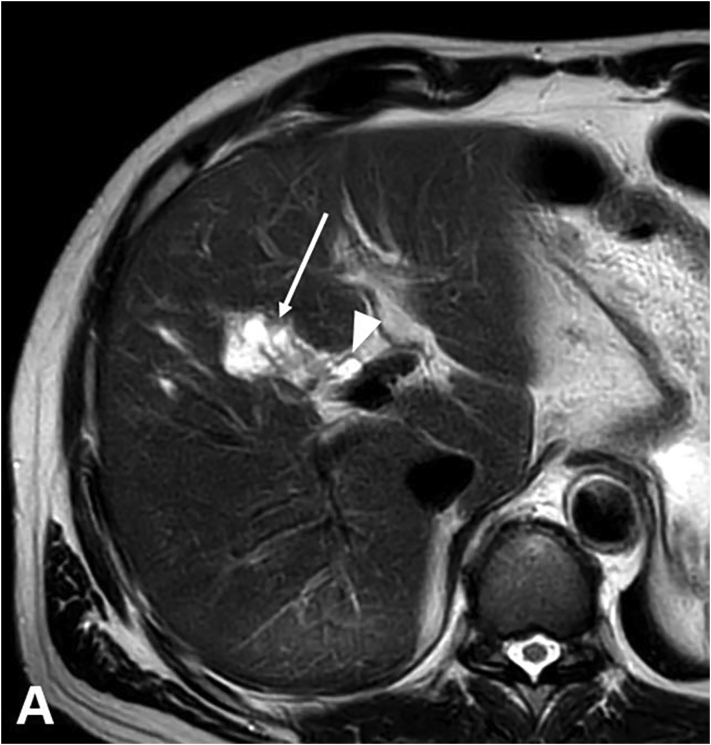
Axial T2 weighted image of the liver at initial MRI showing the subcapsular cystic lesion in liver segment V (arrow) and a normal common hepatic duct (arrowhead).

**Fig. 2:**
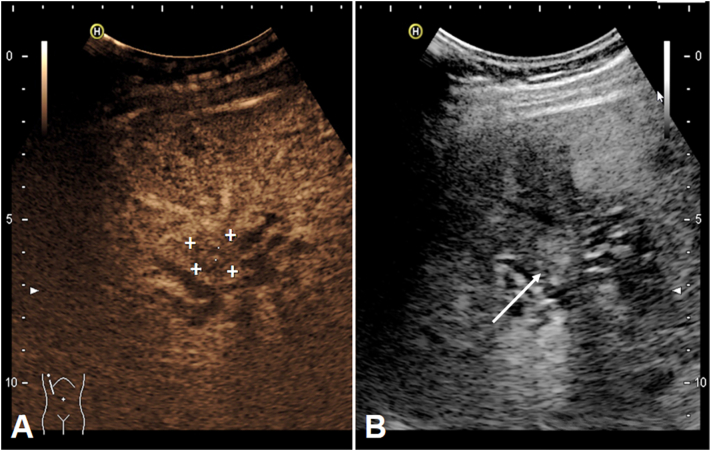
(A) CEUS showing a central lesion of 1.7 cm in diameter (marked with crosses) with weak venous washout between liver segments V and VIII. (B) Conventional sonographic imaging showing the lesion (arrow) and dilated bile ducts distal of the lesion.

As an incidental finding the MRI detected a subcapsular cystic lesion of 2.8 cm in size between the liver segments V and VIII (Fig. 1). The lesion showed no signs of malignancy and remained stable over two years. However, after 3 years a slight increase in size was noted as well as an alteration of the perfusion of the liver segments V and VIII. The bile ducts in these segments were slightly dilated. Malignancy was suspected, which was supported by contrast-enhanced sonography (CEUS) findings showing a lesion in the liver hilus of 1.7 cm in diameter with dilation of the adjacent bile ducts (Fig. 2). Laboratory findings showed an elevated CA 19–9 at 41.3 U/ml (cut-off <27 U/ml), CEA and alpha1-fetoprotein were both normal. There were no indications for cholestasis. Serologic markers for echinococci were negative.

**Fig. 3:**
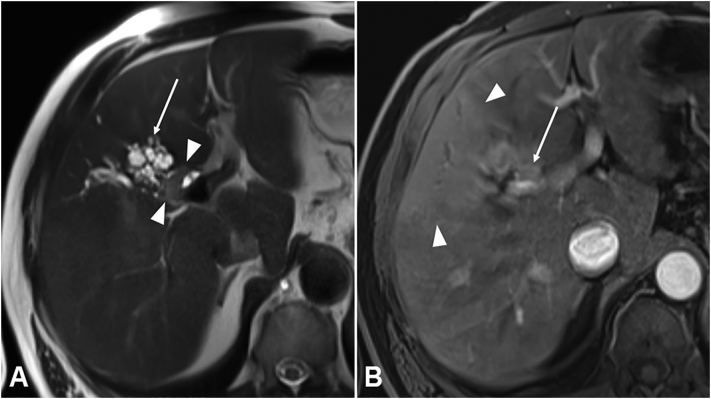
(A) Axial T2 weighted image of follow up MRI with MRCP showing the cystic lesion in liver segment V with a new soft tissue component of 1,7 cm (arrowheads) adjacent to the right hepatic duct. (B) Axial T1 weighted fat suppressed image in the arterial phase after intravenous administration of a gadolinium based contrast agent (gadoxetate disodium, Primovist ™, Bayer Healthcare Pharmaceuticals) demonstrating altered perfusion of liver segment V (arrowheads), dilated bile ducts in liver segment V and VIII and moderate compression of the middle portal vein (trifurcation of the portal vein as anatomic variant) due to the soft tissue lesion in Segment V (arrow).

Magnetic resonance cholangiopancreaticography (MRCP) showed a slight increase of the subcapsular cystic lesion in size and - in correlation to the sonographic findings - an intrahepatic lesion of 1.7 cm was detected on the border between the liver segments V and VIII with compression of the bile ducts, suspicious for a cholangiocarcinoma (Fig. 3). Endoscopic retrograde cholangiopancreaticography (ERCP) was performed to gather histologic samples, cholangioscopic findings showed a slight impression of the right hepatic duct but no further intraluminal findings were described. Cytologic analysis was non-diagnostic with only few normal cylindrical cells and no proof of malignant cells.

Due to the location and the above described negative cytologic findings histological confirmation of the suspected malignant tumour was not possible. The case was discussed at the interdisciplinary tumorboard and right hepatectomy was recommended. Liver function was sufficient for a right hepatectomy as assessed via LiMAx® Test (Humedics GmbH, Berlin, Germany) with a measured value at 492 μg/h/kg (reference value for a normal liver function: >315 μg/h/kg).

### 2.3 Intervention

**Fig. 4:**
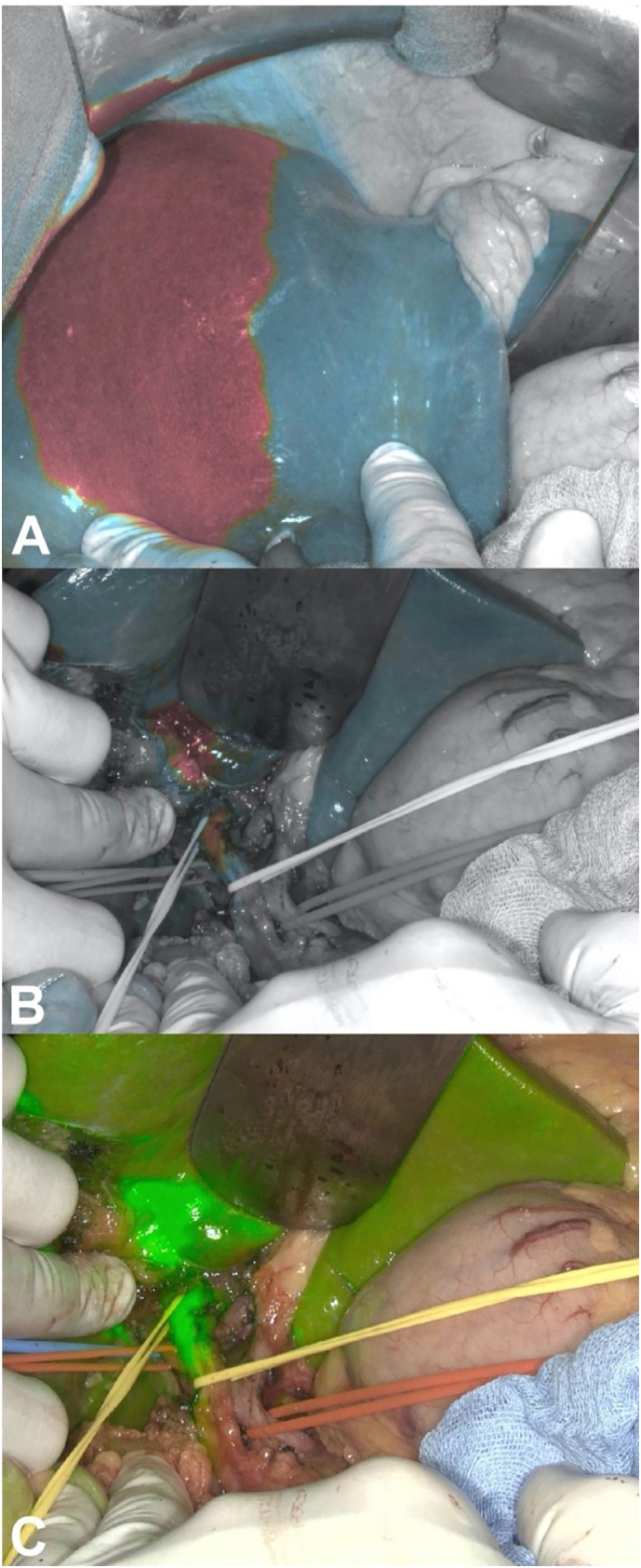
(A) Intraoperative visualization of altered hepatic perfusion in the cholestatic liver segments by ICG staining and Stryker/NOVADAQ SPY-PHI in Colour Segmented Fluorescence Mode (CSF). (B) Identification of the liver tumour in the hilus (CSF mode) and (C) overlay mode.

According to most recent recommendations by Chen et al. [7] 10 mg ICG (VERDYE®, Diagnostic green GmbH, Aschheim-Dornach, Germany) was administered intravenously 12 h before surgery. A standard open right hepatectomy with lymph node dissection of the hepatoduodenal ligament was performed, the intraoperative course was uneventful. After cholecystectomy the tumour could clearly be visualized with a Stryker/NOVADAQ SPY-PHI portable handheld imaging system (Stryker, Kalamazoo, MI 49002, USA) (Fig. 4a–c).

### 2.4 Follow-up and outcome

The postoperative course was uneventful and the patient discharged nine days after the operation with a normal length of hospital stay according to the local diagnosis related groups system (SwissDRG).

Histologic analysis confirmed the diagnosis of a well-/poorly differentiated cholangiocarcinoma (pT2) with two lymph node metastases (pN1). The interdisciplinary board recommended an adjuvant chemotherapy with capecitabine over 6 months in accordance to the BILCAP trial [15].

## 3 Discussion

With the here presented case we demonstrate that confirming a suspicion of intrahepatic cholangiocarcinoma requires a complex diagnostic workup which maybe time consuming and expensive. In this case, histologic/cytologic samples could not be acquired due to the location of the tumour. The lesion could be localized via MRI but translation into the intraoperative was expected to be difficult. Exact determination of tumour margins is of outmost importance to ensure complete resection with negative resection margins. This improves the outcome after resection of hepatic tumours both primary and metastatic. Intrahepatic cholangiocarcinoma is an aggressive entity with a rising incidence. If not found incidentally first diagnosis usually occurs in advanced disease.

This case supports current research that fluorescence guided imaging during hepatic surgery is a safe, easily implementable and cheap method to visualize superficial intrahepatic tumours.

## Informed consent

Written informed consent was obtained from the patient for publication of this case report and accompanying images during pre-operative consultations. A copy of the written consent is available for review by the Editor-in-Chief of this journal on request.

## Sources of funding

No external funding.

## Ethical approval

Exempt from ethical approval.

## Registration of research studies

No “First in Man” report.

## Guarantor

1- Till M. Hempfing
2- Thomas Steffen

## CRediT authorship contribution statement

1- T. Hempfing: conceptualization, data curation, writing - original draft preparation
2- D. Husarik: resources, data curation, writing – reviewing
3- T. Steffen: idea, conceptualization, supervision, writing – reviewing and editing

## Declaration of competing interest

None to declare.
